# Facile Construction of Advanced 1D Metal-Organic Coordination Polymer for Efficient Lithium Storage

**DOI:** 10.3390/molecules28247993

**Published:** 2023-12-07

**Authors:** Jia Du, Xueguo Liu, Bingke Li

**Affiliations:** 1 School of Biology and Chemical Engineering, Nanyang Institute of Technology, No. 80, Changjiang Road, Nanyang 473004, China; huanliu1987@126.com (X.L.); libingke86@126.com (B.L.); 2 Key Laboratory of Advanced Energy Materials Chemistry (Ministry of Education), College of Chemistry, Nankai University, Tianjin 300071, China

**Keywords:** coordination polymer, lithium-ion batteries, anode materials, lithium storage

## Abstract

Recently, coordination polymers (CPs) have been frequently reported in the field of energy storage as electrode materials for lithium-ion batteries (LIBs) due to their highly adjustable architectures, which have a variety of active sites and obviously defined lithium transport routes. A well-designed redox-active organic linker with potential active sites for storing lithium ions, pyrazine-2,3-dicarboxylate (H_2_PDA), was applied for generating CPs by a simple hydrothermal method. When employed as anode materials in LIBs, those two one-dimensional (1D) CPs with an isomorphic composition, [M(PDA)(H_2_O)_2_]_n_ (M = Co for Co-PDA and Ni for Ni-PDA), produced outstanding reversible capacities and stable cycling performance. The Co-PDA displays a substantial reversible capacity of 936 mAh g^−1^ at 200 mA g^−1^ after 200 cycles, as well as an excellent cycling life at high currents. According to the ex situ characterizations, the high reversible specific capacity of the post-cycled electrodes was found to be a result of both the transition metal ions and the organic ligands, and Co-PDA and Ni-PDA electrode materials show reversible insertion/extraction processes that are accompanied by crystallization to an amorphous state.

## 1. Introduction

In order to alleviate the progressive exhaustion of fossil fuels and the consequent climate issues, the use and development of sustainable energy sources (including solar, geothermal, wind and biomass) are a global trend, which has prompted society to develop in an environmentally sustainable manner [1]. The efficient and reasonable use of renewable energy cannot be achieved without electrochemical energy storage systems, and the development of high-efficiency energy storage devices has become a key hot spot in the world [2]. Lithium-ion batteries (LIBs) have emerged as one of the most attractive power storage devices of energy and electric vehicles due to their large energy capacity, good environmental compatibility, and lack of memory effect during cycling [3]. The commonly inorganic electrodes used in LIBs can provide high capacities, but they usually contain heavy metal elements, and these problems such as capacity attenuation and poor cycle stability limited their practical application [4,5].

In contrast, organic electrode materials, particularly some small molecules or polymers with high redox activity, have the potential to replace inorganic materials owing to the significant advantages of high theoretical specific capacity, versatility, and sustainability. Despite these advantages, organic compounds typically have problems with dissolution and low electronic conductivity in electrolytes. Polymerization has been shown to help reduce the dissolution of organic materials in electrolytes; however, this may lead to a low theoretical capacity as the unit weight absorbed by each electron inevitably increases [6].

Coordination compounds, which are constituted by an infinite number of coordination bonds between metal ions and organic ligands, have received a lot of attention due to their unique abilities in the fields of optics, magnetism, electronics and catalysis [7,8]. By combining organic bridging ligands with redox activity with metal centers to construct coordination compounds, the lithium storage capacity of organic materials can be increased, as both metal centers and organic ligands have the potential to be employed as lithium storage sites. At the same time, the solubility of organic components can be inhibited by the formation of coordination bonds, and the diverse structural composition provides a clear pathway for the transport of lithium ions [9].

As a branch of coordination compounds, the coordination polymers (CPs) with redox-active organic ligands or metal centers have recently acquired popularity as electrode materials. Cheng et al. stated in 2016 that the 1D CP [Co_1_._5_L(H_2_O)_4_]_n_ (H_3_L = 4-hydroxypyridine-2,6-dicarboxylic acid) can be employed as an anode material of LIBs, with a reversible capacity of 431 mAh g^−1^ at 50 mA g^−1^ after 50 cycles [10]. By connecting the mononuclear complex to a 1D CP, Du et al. demonstrated that the lithium storage capacity was dramatically increased, going from 554 to 1025 mAh g^−1^ at 100 mA g^−1^ [11]. The one-dimensional CP coordination chain structure has a favorable lithium-ion diffusion channel, as demonstrated by both experimental data and ab initio MD simulations. Co-HIPA and Ni-HIPA (H3IPA = 5-hydroxyisophthalic acid) are two isostructural 1D CPs with high electrolyte stability that have been employed as anodes for LIBs. Co-HIPA demonstrated exceptional lithium storage capability with a capacity of 1043 mAh g^−1^ at 200 mA g^−1^ for 200 cycles because of its great coordination ability and quick lithium storage kinetics [12]. Compared with previously reported 3D MOF electrodes, the structure of 1D chains connected by weak hydrogen bonds ensures that the material can fully expose the redox-active site to lithium-ion binding and thus allows the materials to approach their maximum potential capacities. Additionally, in these reported CPs-based or MOFs-based electrode materials, carboxylate groups (such as 1,3,5-benzenetricarboxylate and 1,4-benzenedicarboxylate) are frequently employed to coordinate with various metal centers due to the electron-donating function of the carboxylate group and the benzene ring in storing lithium ions. CPs or MOFs with other kinds of organic linkers are rarely used as electrode materials.

In this contribution, applying pyrazine-2,3-dicarboxylate (H_2_PDA) as the organic ligand, the two 1D CPs, namely [M(PDA)(H_2_O)_2_]_n_ (M = Co for Co-PDA and Ni for Ni-PDA), were synthesized and their Li^+^ storage performance were studied. The H_2_PDA utilized the unsaturated carbonyl group and pyrazine as the redox-active sites, and uses the coordination of the N and O in the carbonyl group and pyrazine group with metal ions to realize the reversible storage of lithium ions. Through changing the metal center, lithium storage performance is greatly changed between Co-PDA and Ni-PDA due to enhanced reaction kinetics and active site utilization. The Co-PDA can demonstrate a significant capacity of 936 mAh g^−1^ at 200 mA g^−1^ after 200 cycles when used as the anode of LIBs for the first time. It also displays an excellent cycling life at the high currents, while a charge capacity of 670 mAh g^−1^ is retained after 200 cycles at 200 mA g^−1^ for the Ni-PDA. The mechanism of lithium storage studied by the ex situ measurements demonstrated that the synergistic lithium storage of metal centers and the carboxylate groups and pyrazine rings of organic ligands are essential for maintaining the high lithium storage properties.

## 2. Results

**Figure 1:**
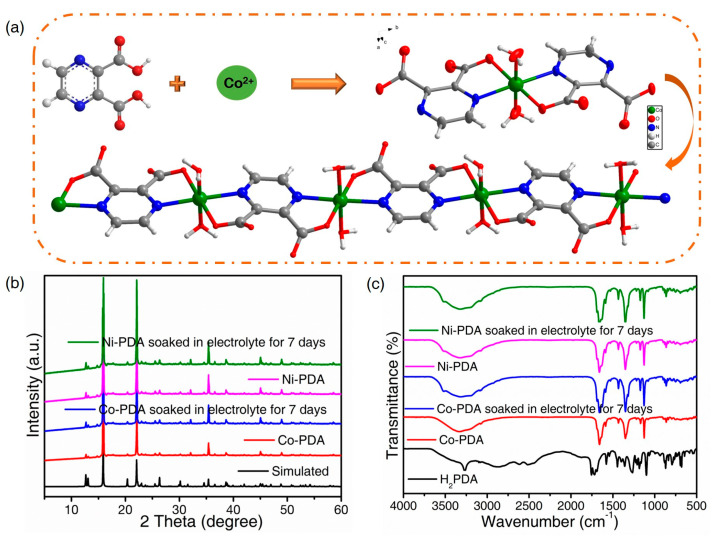
(**a**) The structural composition of Co-PDA; PXRD patterns (**b**) and FTIR spectra (**c**) of Co-PDA and Ni-PDA before and after soaked in electrolyte.

According to single-crystal X-ray diffraction research, Co-DPA crystallized in the orthorhombic system with the space group Pcca and unit cell volumes of 1739.34 Å^3^ (Table S1) [13]. The asymmetric unit consists of one Co(II) ion, one fully deprotonated H_2_PDA unit and two coordinated H_2_O molecules. Each Co(II) ion forms a deformed octahedral structure around the Co(II) core when it connects to four oxygen (Co-O = 2.049(2)-2.077(2) Å) and two nitrogen atoms (Co-N = 2.151(2) and 2.1577(9) Å) (Table S2). Two water molecules and two PDA^2−^ unit linker carboxylate groups each supply two oxygen atoms, whereas two pyrazine rings and two PDA^2−^ unit linker units each contribute two nitrogen atoms. A deformed square planar grid is produced around the Co(II) center as a result of the PDA^2−^ linker’s symmetric bonding to two Co(II) centers. The chain structure is formed by the alternating linkage of PDA^2−^ and metal centers (Figure 1a), and a three-dimensional supramolecular framework is formed by neighboring chains being connected by hydrogen bonds between H_2_O molecules and the oxygen of ligands (Table S3 and Figure S1).

The powder X-ray diffraction (PXRD) (Figure 1b) and Fourier transform infrared spectroscopy (FTIR) (Figure 1c) characterizations were used to verify the phase purity of Co-PDA and Ni-PDA. PXRD patterns of the obtained Co-PDA and Ni-PDA are almost identical to those of the simulated Co-PDA crystal structure, confirming a pure solid-state phase and isostructural composition. Furthermore, Co-PDA and Ni-PDA were soaked in the electrolyte for seven days to test their electrolyte stability, and the CPs preserve their structural integrity after immersion [14].

The positions and Intensities of the peaks in the FTIR spectra are almost identical for the two CPs, and Co-PDA and Ni-PDA can keep their structural integrity after being soaked in the electrolyte. The -OH stretching vibrations of the coordination water molecules produce a strong and broad band between 3000 and 3400 cm^−1^ [15]. Compared with the organic ligands, no absorption peak was found in the range of 1690–1720 cm^−1^ (υ_c=o_ of COOH) and 3266 cm^−1^ (δ_O-H_ of COOH), indicating the carboxylic acid group of H_2_PDA has been deprotonated; the strong absorption peaks at 1584 and 1379 cm^−1^ belong to the asymmetric stretching vibration and symmetric stretching vibration of the carboxyl group, respectively [16]. The difference of asymmetric stretching and symmetric stretching vibration is greater than 200 cm^−1^, indicating the carboxyl group was coordinating to the metallic ion in monodentate fashion. The characteristic peak of the N=C bond moves from 1579 cm^−1^ to a high wave number of 1650 cm^−1^, indicating that nitrogen atoms participate in the coordination (Figure S2) [17].

The thermogravimetric analysis (TGA) of Co-PDA and Ni-PDA were observed at the temperatures ranging from 40 to 800 °C (Figure S3). Co-PDA loses 13.77% of its weight from 80 to 305 °C, while Ni-PDA loses 13.56% from 95 to 312 °C, indicating the release of two coordinated water molecules (calcd 13.78% for Co-PDA and 13.80% for Ni-PDA). Further heating resulted in rapid weight losses, indicating that the frameworks had collapsed. Scanning electron microscopy (SEM) was used to analyze the morphologies of Co-PDA and Ni-PDA (Figure S4), which revealed that they are block-shaped with diameters ranging from 50 to 120 μm, respectively. The corresponding EDS mapping images suggest that C, N, O, Co and C, N, O, Ni were homogeneously dispersed over the entire sample of Co-PDA and Ni-PDA.

**Figure 2:**
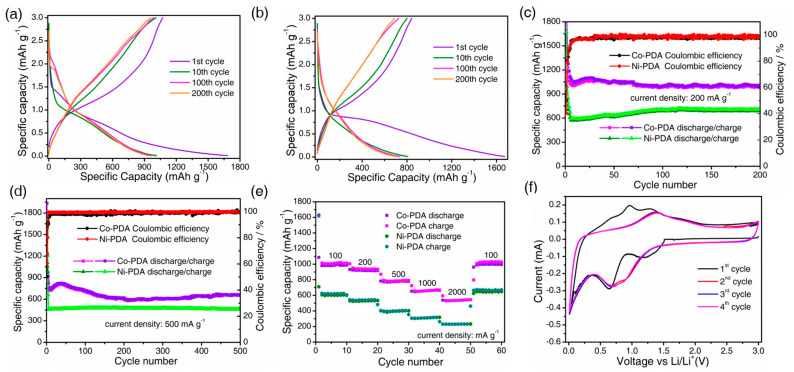
Electrochemical properties of Co-PDA and Ni-PDA: Galvanostatic charge–discharge curves of (**a**) Co-PDA and (**b**) Ni-PDA; cycling stability at (**c**) 200 mA g^−1^, (**d**) 500 mA g^−1^, rate performance (**e**) and cyclic voltammetry curves (**f**).

The electrochemical performance of the two isostructural Co-PDA and Ni-PDA as LIBs anode materials was investigated in a voltage window of 0.01–3.0 V (vs. Li/Li^+^). The initial discharge and charge capacity of Co-PDA are 1682 and 1071 mAh g^−1^ at a current density of 200 mA g^−1^, with an initial Coulombic efficiency (ICE) of 63.6% (Figure 2a). The first irreversible capacity loss is due to the irreversible processes including the formation of solid electrolyte interphase (SEI) layers and interfacial lithium storage [18]. Furthermore, the generation of Li_2_O as a result of the reaction between coordinated water molecules in Co-PDA and lithium causes the reversible capacities degradation from the second cycle to tenth cycle. As for Ni-PDA, the initial discharge and charge capacity are 1673 and 843 mAh g^−1^ while the ICE is only 50.43% (Figure 2b). As shown in Figure 2c, the Co-PDA and Ni-PDA demonstrated impressive cycling stability and superb reversible capacity at the current density of 200 mA g^−1^. The discharge capacities of Co-PDA and Ni-PDA still retain at 936 and 612 mAh g^−1^ after 200 cycles, which is greater than the theoretical capacity of graphite (372 mAh g^−1^). The reversible capacities of Co-PDA and Ni-PDA after 500 cycles at 500 mA g^−1^ are 662 and 460 mAh g^−1^, respectively (Figure 2d). When performed at 1000 mA g^−1^, the reversible capacities of Co-PDA and Ni-PDA are 553 and 360 mAh g^−1^ after 1000 cycles, respectively (Figure S5).

Figure 2e depicts the rate performance of the electrodes. When the rates changed from 100 to 200, 500, 1000 and 2000 mA g^−1^, the corresponding discharge capacities are 1013, 938, 781, 664 and 531 mAh g^−1^ for Co-PDA and 612, 538, 403, 307 and 231 mAh g^−1^ for Ni-PDA, respectively. Reversible capacities quickly return to their initial stage when the current rate is reduced to 100 mA g^−1^, showing excellent cycle stability. In contrast to the organic ligand H_2_PDA (Figure S6), Co-PDA and Ni-PDA exhibit a high reversible capacity and favorable cycling stability.

Cyclic voltammetry (CV) curves were obtained in the voltage from 0.01 V to 3 V at a rate of 0.1 mV s^−1^ (Figure 2f). Due to the generation of SEI, the irreversible cathodic peak for Co-PDA is around 1.3 V in the initial cathodic scan [10,19]. The reduction peaks at 0.7 and 1.1 V are attributed to the insertion of lithium ions with organic ligands and the conversion of Co^2+^ to Co^0^, respectively, and the reverse lithium-ion extraction process can be observed with the oxidation peaks at 1.3 V and 1.8 V [20,21]. The CV curves overlapped from the second to the fifth cycle, demonstrating the strong reversibility and stability of the electrochemical reactions. Similarity to Co-PDA, Ni-PDA exhibits two-step lithiation–delithiation processes after the first cycle. The reduction peaks at around 1.32 and 0.85 V and oxidation peaks at around 1.75 and 1.28 V are attributed to the binding/extraction reaction of Li^+^ ions with organic ligands and the redox reaction between Ni^2+^ and Ni^0^, respectively (Figure S7) [22,23].

**Figure 3:**
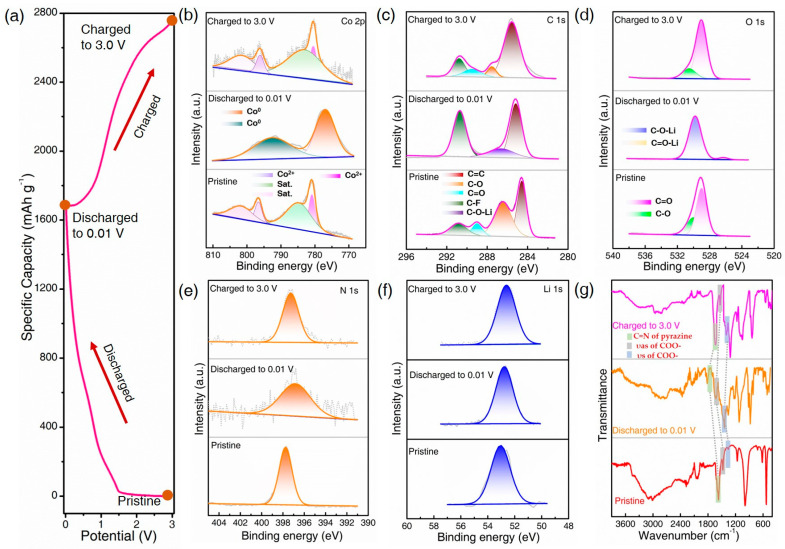
Electrochemical reaction mechanism of Co-PDA: (**a**) the selected states of the discharge/charge profile; the XPS spectra of (**b**) Co 2p, (**c**) C 1s, (**d**) O 1s, (**e**) N 1s and (**f**) Li 1s; (**g**) FTIR spectra.

To completely understand the redox reaction mechanism of Co-PDA, the states highlighted in the second charge–discharge profile shown in Figure 3a were chosen for further investigation. X-ray photoelectron spectroscopy (XPS) was used to track the structural evolution of the Co-PDA electrode reacting with lithium. As presented in Figure 3b of the Co 2p XPS spectrum, the main peaks at 780.2 and 795.7 eV and the satellite peaks at 785.5 and 804.2 eV in the pristine state are assigned to Co^2+^ [24]. When further discharged to 0.01 V, the two main peaks moved to 777.7 and 792.8 eV, and the satellite peaks disappeared, illustrating that Co^2+^ transformed to Co^0^ [25]. In the charging process, the main peaks moved to 780.2 and 795.7 eV, accompanied by the reappearance of the satellite peak at 785.5 and 804.2 eV of Co^2+^, suggesting the oxidation from Co^0^ to Co^2+^. In comparison to the pristine state C 1s spectra at 286.4 of C-O and 284.8 eV of the C=C bonds, the peak at 288.7 eV of the C=O bond disappears, and the peak of the C–O bond shifts to 285.8 eV upon lithium ion insertion (Figure 3c) [26]. The peaks of C=O and C–O bonds recover after the full charge process. The peaks at 528.9 and 530.1 eV in Figure 3d of the O 1 s spectrum can be assigned to the C=O bond and the C–O bond in pristine state. The signal of the C=O bond vanished upon discharge, and a new signal forms at 529.8 eV and 526.5 eV, which is due to the development of the O-Li bond [27]. The peak of the C–O and C=O bonds can be detected when charged, illustrating the reversible uptake and extraction of lithium ions in the organic ligands. When discharged, the peak in the N 1s spectrum that was at 398.7 eV moved to a lower binding energy at 397.8 eV as a consequence of absorbing electrons and bonding with lithium (Figure 3e), and it could be reversible back to the higher binding energy when charged [28]. Because of the generation of the SEI, some inserted Li are presented in the pristine condition in the Li 1 s XPS spectrum, and the intensity of Li 1 s gradually increased when discharged and decreased when charged (Figure 3f) [29]. The XPS spectra of Ni-PDA electrodes shift in a similar way to that of the Co-PDA electrodes (Figure S8).

Ex situ FTIR studies were carried out after complete lithiation and delithiation to gain a better understanding of the redox reaction of the organic ligand and further investigate the storage mechanism of lithium ions (Figure 3g). The vibrations of asymmetric stretching and symmetric stretching of the carboxyl group shift to higher frequencies (1608 and 1407 cm^−1^) when discharged to 0.01 V, revealing that the electron density has increased [30], and they can return to low frequencies when charged to 3.0 V, demonstrating that the redox processes of carboxyl groups are extremely reversible. The vibrational modes of C=N bonds at 1650 cm^−1^ vanish and reappear accompanied by the lithiation–delithiation process, and the peak at 1735 cm^−1^ induced by the Li−N−C vibration mode emerged when discharging but vanished after recharging to 3 V, indicating that nitrogen undergoes a reversible lithiation–delithiation process [31]. The carboxylate groups and pyrazine rings of the organic ligands undergo a highly reversible redox reaction, as evidenced by the shapes of the FTIR peaks changing noticeably during the discharge process but nearly returning to their original appearances during the subsequent charge process.

**Figure 4:**
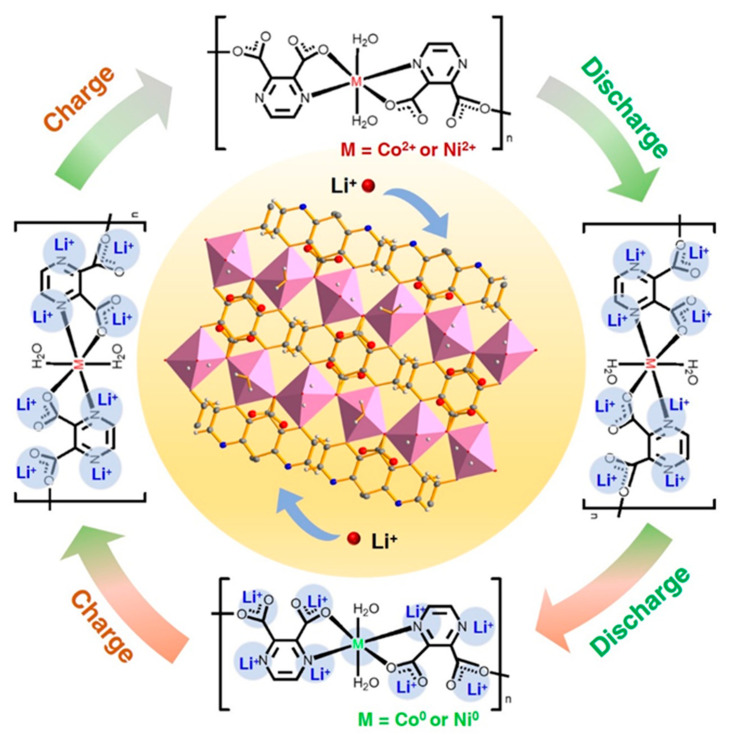
Schematic diagram of the lithiation–delithiation processes of the two CPs.

The changes in the FTIR spectra of Ni-PDA electrodes are comparable to that of the Co-PDA electrode and presented in Figure S9. According to Figure S10, the FTIR peaks of the Co-PDA and Ni-PDA electrode in the selected states are reversible changed, demonstrating the coordination environments of Co-PDA and Ni-PDA are maintained throughout cycling. Results from the CV, XPS and FTIR analysis indicated that the metal center and organic ligands in the CPs could both be combined with lithium ions, and the presumed lithium storage process is depicted in Figure 4.

Ex situ PXRD characterization of Co-PDA and Ni-PDA is conducted at the pristine state, after one cycle and after two cycles of full lithiation and delithiation (Figure S11). There are no obvious diffraction peaks, indicating that the structure had lost its long-range order and become amorphous [32]. The amorphized active materials possess more isotropic ion diffusion channels without grain boundaries, which is advantageous for exposing redox-active sites and promoting the migration of lithium ions [33,34]. Because of the reversible generation of coordination units and structural stability, the Co-PDA and Ni-PDA electrodes show steady lithium storage performance throughout cycling.

**Figure 5:**
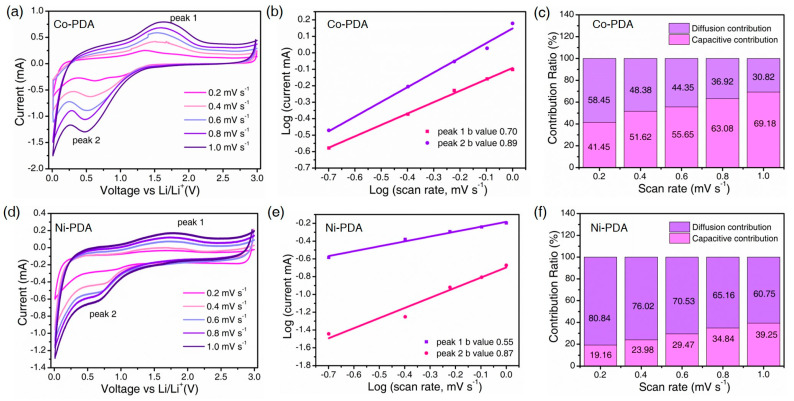
Electrochemical kinetics of Co-PDA (**a**–**c**) and Ni-PDA (**d**–**f**): (**a**,**d**) CV curves at different scan rates; (**b**,**e**) the relationship between the peak currents and scan rates; (**c**,**f**) percentage of diffusion and capacitive contributions.

The stepped CV curves at different scan rates (between 0.2 and 1.0 mV s^−1^) are conducted to further analyze the charge/discharge reaction kinetics of Co-PDA (Figure 5a–c) and Ni-PDA (Figure 5d–f). The Co-PDA was described in detail as an example to reveal the whole process. The intensity of the anodic and cathodic peaks on the CV curves grow as the scan rate rises, demonstrating the extraordinary reversibility (Figure 5a). A qualitative analysis of the capacitive effect can be obtained according to the relationship between the measured current (*i*) and scan rate (*v*) (a and b both are constants) [35]:

(1) $$i={av}^{b}$$

The power factor b as the slope can be acquired by fitting between log(*v*) and log(*i*) linearly. When the value of b reaches 0.5, the ion diffusion process takes over the electrode reaction, and when it approaches 1.0, the capacitance process controls the entire reaction process [36]. As shown in Figure 5b, the b values of Co-PDA are 0.89 and 0.70 during the redox reaction, respectively, demonstrating that both capacitive and diffusion processes contribute to lithium storage. Additionally, the following equation can be used to quantify the capacitance contribution:

(2) $$i=k_{1}v+k_{2}v^{1/2}$$

where the contributions from capacitance and diffusion are denoted by *k*_1_*v* and *k*_2_*v*^1/2^, respectively. As shown in Figure 5c, the capacitance behavior contributed 41.45%, 51.62%, 55.65%, 63.08% and 69.18%, respectively, when the scan rate ranges from 0.2 to 1.0 mV s^−1^. The increased capacitive contribution is advantageous for the rapid transfer of Li^+^, guaranteeing the enhanced rate performance of Co-PDA. The comparable test was also carried out on Ni-PDA, as shown in Figure 5d–f. The intensities for anodic and cathodic peaks increase with the scan rate increases, illustrating that the Ni-PDA electrode possesses an exceptional reversibility. The b values are calculated as 0.87 and 0.55 for the oxidation and reduction process, respectively, and the corresponding capacitance contribution of Ni-PDA increased from 19.16%, 23.98%, 29.47%, 34.84% to 39.24%. In comparison to Ni-PDA, the b values and the capacitive contribution are higher in Co-PDA with different scan rates, which enables the better cycle stability and rate performance than Ni-PDA.

For a better comparison of the diffusion of Li^+^ for Co-PDA and Ni-PDA during cycling, the Li diffusion coefficients (D_Li+_) are calculated by the galvanostatic intermittent titration technique (GITT) (Figure S12) [37]. Calculated by GITT, Co-PDA possesses an obvious higher D_Li+_ (3.5 × 10^−14^~8.8 × 10^−14^ cm^2^ s^−1^) than Ni-PDA (8 × 10^−15^~4.3 × 10^−14^ cm^2^ s^−1^) at different states, further proving that the structure of Co-PDA can facilitate Li^+^ diffusion more effectively than Ni-PDA.

**Figure 6:**
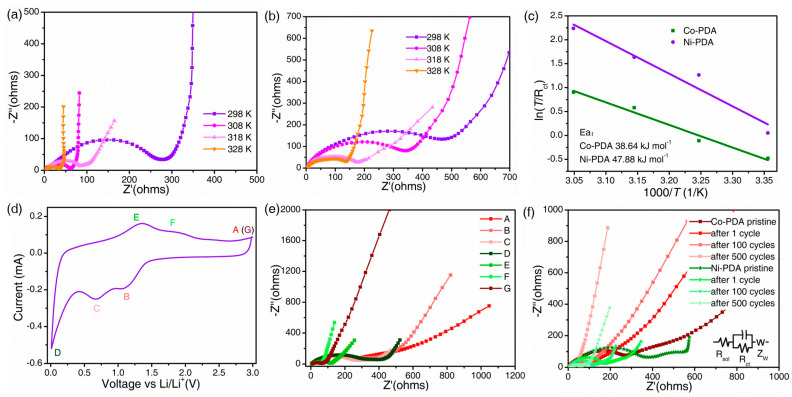
EIS of (**a**) Co-PDA and (**b**) Ni-PDA at different temperatures; (**c**) Arrhenius plots of Co-PDA and Ni-PDA; (**d**) CV curves of Co-PDA (A 3.0 V, B 1.23 V, C 0.68 V, D 0.01 V, E 1.35 V, F 1.82 V and G 3.0 V) and the corresponding impedance spectra (**e**); (**f**) Nyquist plots of Co-PDA and Ni-PDA in the states of pristine, after 1, 100 and 500 cycles.

The electrochemical impedance spectroscopy (EIS) measurements were taken at different states to better understand the kinetics of Co-PDA and Ni-PDA. The charge transfer impedance (*R_ct_*) and ion diffusion process are represented as a semicircle in the high-frequency area and a straight line in the low-frequency region of the EIS spectrum, respectively. The EIS of Co-PDA and Ni-PDA during the initial charge process is evaluated at various temperatures to derive the activation energy, as illustrated in Figure 6a,b. The *R_ct_* steadily decreases as the temperature rises from 298 K to 328 K, and the *R_ct_* of Co-PDA (Figure 6a) is consistently lower than that of Ni-PDA (Figure 6b) at the same temperature, indicating the rapid reaction kinetics of Co-PDA. The following formula can be used to calculate the apparent activation energy (*E_a_*) [38]:

(3) $$i_{0}=RT/nFR_{ct}$$

(4) $$i_{0}=Ae^{-E_{a}/RT}$$

where *i*_0_, *A*, R, *T*, *n* and F represent the exchange current, the temperature independent coefficient, the gas constant, the absolute temperature, the number of transferred electrons and the Faraday constant, respectively. The fitting results indicated the *E_a_* values of 38.64 and 47.88 kJ mol^−1^ for the Co-PDA and Ni-PDA, respectively (Figure 6c). The lower *E_a_* value of Co-PDA indicates the faster reaction kinetics, which is conducive to the realization of faster rate charge transfer, higher reversible capacity and superior rate performance.

Furthermore, EIS were carried out in chosen states to investigate the change in Co-PDA throughout the charge/discharge processes. The change in EIS during the first cycle confirmed the formation of the SEI layer and the irreversible lithiation process (Figure 6d) [39]. When discharged to 1.23 V, the *R_ct_* expands, indicating that lithium-ion intercalation is occurring in Co-PDA. Due to the formation of the SEI layer on the interface, the *R_ct_* grows dramatically when discharged to 0.01 V. When charged to 1.35 V, the *R_ct_* decreases due to the breakdown of the SEI layer induced by the volume change during the lithium-ion diffusion process. When fully charged to 3.0 V, the *R_ct_* shows a substantial decrease, indicating that the impedance gradually decreases after the cycle (Figure 6e) [40]. The changes in EIS at selected states of Ni-PDA electrodes are comparable to that of the Co-PDA electrode (Figure S12a,b).

The EIS were analyzed at various cycles to observe the variation in reaction kinetics (Figure 6f). The fitting circuit diagram of the change process is embedded in Figure 6f. In detail, the intercept point of the high frequency region and the real axis corresponds to the solution resistance *R_sol_*, and the semi-circle of the low-frequency region corresponds to parallel action of the charge transfer resistance (*R_ct_*). The low-frequency region is a straight line associated with the diffusion process, which can be represented by the Warburg impedance Z_w_. In the subsequent cycles, there was an ever-declining trend in *R_ct_*, which can be observed from 272 Ω at pristine to 40 Ω at the 500th cycle of Co-PDA and from 374 Ω at pristine to 58 Ω at the 500th cycle of Ni-PDA (Table S4). The decreased *R_ct_* values of Co-PDA and Ni-PDA show that these electrode materials have been fully activated, which is favorable for the lithium storage performance [41]. Compared with Ni-PDA, the *R_ct_* of Co-PDA was significantly lower in the same cycles, demonstrating that the Co-PDA electrode could greatly speed charge transfer and promote increased Li^+^ diffusion kinetic behavior [42]. We have also calculated the D_Li_^+^ base on the EIS results (Figure S12c–e). The D values (×10^−14^ cm^2^ s^−1^) are calculated as 1.59, 4.91, 6.82 and 8.63 for Co-PDA at the pristine, after 1 cycle, after 100 cycles and after 500 cycles, respectively, and they were 1.21, 2.38, 4.12 and 5.56 for Ni-PDA at the corresponding states. The diffusion coefficient based on the EIS of Co-PDA is larger than that of Ni-PDA, which is close to the result from GITT, implying the higher diffusion rate of Co-PDA. Furthermore, the calculated electrical conductivities of Co-PDA (4.86 × 10^−5^~3.29 × 10^−4^ S cm^−1^) are higher than Ni-PDA (3.54 × 10^−5^~2.25 × 10^−4^ S cm^−1^) at different states (Figure S12f), which is a benefit to obtaining the high lithium storage performance of Co-PDA.

Obviously, the superior performance of LIBs can be obtained by the chain-based supramolecular structure, which is not constrained by the channel size and can effectively promote lithium-ion diffusion. The Co-PDA electrode is preferable to the Ni-PDA electrode because: (1) the calculated b values of the anodic and cathodic peaks for Co-PDA (0.89, 0.70) indicate a higher surface-controlled capacitive contribution to total capacity as compared to Ni-PDA (0.87, 0.55), enables the better cycle stability and rate performance; (2) the higher D values of Co-PDA than that of Ni-PDA proving that Co-PDA is more favorable for improving Li^+^ diffusion; (3) the lower *E_a_* value and EIS results at different states of Co-PDA indicates the faster reaction kinetics, facilitating the charge transfer at a high rate and benefiting for the high reversible capacity and superb rate capability. The synergistical redox reactions on both metal centers and the unsaturated carbonyl group and pyrazine in the organic moieties play a crucial role in the lithium insertion/extraction process and enhance the electrochemical performance. Co-PDA and Ni-PDA have lost long-range order within the structure and are all largely amorphous after cycles, and the locally remaining coordination structure demonstrate a superior redox-active performance.

## 3. Materials and Methods

### 3.1. Materials Characterization

Measurements of powder X-ray diffraction (PXRD) were made using a Rigaku Ultima IV apparatus (Tokyo, Japan) at a scanning rate of 5° min^−1^ and in the 2θ range of 5–60°. Utilizing a Labsys NE-TZSCH TG 209 Setaram apparatus (Zurich, Switzerland) with a heating rate of 10 °C min^−1^ in a nitrogen environment conducted the thermal gravimetric analysis (TGA) experiments. Using a Bruker ALPHA-T infrared spectrophotometer (Karlsruhe, Germany), the Fourier transform infrared (FT-IR) spectra were acquired in the 400–4000 cm^–1^ wavenumber range. A ZEISS MERLIN compact (field emission) scanning electron microscope (Oberkochen, Germany) operating at 5 kV and 100 μA was used to measure the scanning electron microscopy (SEM) images. Using an Al Kα X-ray source, Kratos AXIS Ultra DLD spectrometer (Manchester, UK) was used to record X-ray photoelectron spectroscopy (XPS). Using graphite-monochromatic Mo Kα radiation (λ = 0.71073 Å) at 120 K, an Agilent Technologies Supernova single-crystal X-ray diffractometer (USA) was used to get the crystal structure of Co-PDA. The Olex2 software (Olex 2-1.2) packages’ SHELXS program was used to solve the structure directly, then SHELXL’s full-matrix least-squares techniques were applied to F2 to enhance it. By using a riding model, all of the hydrogen atoms on the organic ligands were refined after being computed geometrically.

### 3.2. Synthesis of Co-PDA and Ni-PDA

[Co(PDA)(H_2_O)_2_]_n_ synthesis: 3 mL of DMF and 9 mL of H_2_O were used to dissolve a combination of pyrazine-2,3-dicarboxylic acid (0.2 mmol) and Co(NO_3_)_2_·6H_2_O (0.2 mmol), which was then agitated at room temperature for 30 min. After the mixture was sealed in a 23 mL stainless steel vessel lined with Teflon, the mixture was heated at 130 °C for 12 h under autogenous pressure and then allowed to cool to room temperature. It was then allowed to cool to ambient temperature. Rod-shaped crystals with a brick red tint were produced. DMF and deionized water were used to wash the crystals in turn. Lastly, the crystals were dried at 55 °C, yielding 63% of the total based on Co^2+^. The synthesis of [Ni(PDA)(H_2_O)_2_]_n_ was carried out in an approach comparable to that of [Co(PDA)(H_2_O)_2_]_n_, with the exception that Ni(NO_3_)_2_·6H_2_O (0.2 mmol) was utilized in replacement of Co(NO_3_)_2_·6H_2_O (0.2 mmol). Crystals featuring a spherical shape and a dark blue color were created (yield = 60% based on Ni^2+^).

### 3.3. Cells Fabrication and Electrochemical Measurements

Half-cells of the standard CR2032 coin type were built in a glovebox filled with argon. The electrode-active materials have been mixed with Ketjen black and polyvinylidene fluoride (60:30:10 in weight) in N-methyl-2-pyrrolidone to create a homogeneous slurry. The resulting slurry was poured over copper foil and vacuum-dried for 24 h at 60 °C. A work electrode has a mass loading ranging from 1.2 to 1.6 mg cm^−2^. Li metal was the counter- and-reference electrode used in the production of lithium-ion batteries. The electrolyte composition consisted of 1 M LiPF_6_ dissolved in a 1:1, *v*/*v* solution of ethylene carbonate (EC) and diethylcarbonate (DEC), and the polypropylene film was used as separators of the cells. Before the electrochemical testing, the batteries were aged for a whole night. Based on the weight of the active materials, galvanostatic charge–discharge experiments were carried out on a LAND battery tester in a temperature-controlled thermotank with a potential range of 0.01–3.0 V (vs. Li/Li^+^). Cyclic voltammetry (CV) and electrochemical impedance spectroscopy (EIS) (frequency range: 0.01–100 KHz) measurements were performed on a CHI 660E electrochemical workstation (ChenHua Instruments Co., Shanghai, China). Newly constructed cells were galvanostatically discharged and/or charged to various cutoff voltages at 100 mA g^−1^ following chosen cycles in order to examine the Li-storage mechanism. Within a glove box filled with Ar, the cycling cells were dismantled and the powders that were scraped were gathered for PXRD, FTIR and XPS analysis.

The Li^+^ diffusion coefficients (D_Li+_) are calculated by GITT method according to the equation:

(5) $$D=\frac{4}{\pi \tau }{\left(\frac{m_{B}V_{M}}{M_{B}S}\right)}^{2}{\left(\frac{\Delta E_{s}}{\Delta E_{t}}\right)}^{2}$$

where *τ*, *m_B_*, *V_M_*, *S* and *M_B_* are the pulse time, the electrode mass of Co-PDA and Ni-PDA, molar volume of active material, the contact area between electrode and electrolyte and the molecular weight of Co-PDA and Ni-PDA, respectively. Δ*Eτ* represents the change in overall voltage during a pulse step that does not include the IR drop, and Δ*Es* is the voltage difference between each step’s steady state and beginning state. The lithium-ion diffusion coefficient based on EIS measurements according to the equation:

(6) $$D=\frac{{R^{2}T}^{2}}{2A^{2}n^{4}F^{4}C^{2}\sigma^{2}}$$

where R is the gas constant; *T* is the test temperature; *A* is the surface area of the electrode; *n* is the number of the electrons per molecule attending the electron transfer reaction; F is the Faraday constant; *C* is the concentration of lithium ion; *σ* is Warburg factor relative to *Z*′, which can be obtained through Equation (7) (as the slope of *Z*′ plotted against *ω*^−1/2^, Figure S12a,b). The electrical conductivity is calculated according to the Equation (8):

(7) $$Z^{\prime }=\sigma \omega^{-\frac{1}{2}}$$

(8) $$\delta =\frac{L}{RS}$$

where *δ* is the electrical conductivity, *L* is the thickness of the electrode, *R* is the imaginary impedance spectroscopy resistance based on EIS measurements and *S* is the cross-section area of the electrode.

## 4. Conclusions

In summary, using a one-pot hydrothermal technique, 1D CPs between M^2+^ (M = Co and Ni) and pyrazine-2,3-dicarboxylate were synthesized. The stable coordination bonds that connect the polymer chains are advantageous for the quick electron transfer and Li+ diffusion. Co-PDA demonstrated improved electrochemical capabilities as anodes for LIBs, including superior cycling stability (936 mAh g^−1^ at 200 mA g^−1^ after 200 cycles) and long cycle stability (553 mAh g^−1^ at 1000 mA g^−1^ after 1000 cycles). Studies on the electrochemical mechanism reveals that synergistical redox processes on both metal centers and organic moieties are vital to the lithiation–delithiation process as well as to the improvement of the electrochemical performance. Similar to the phenyl carboxylic acid ligands used in traditional CPs-based or MOFs-based electrode materials, the unsaturated carbonyl group and pyrazine in the pyrazine-2,3-dicarboxylate with redox properties selected in this paper also contribute to lithium storage properties. This research lays the groundwork for future research into the use of transition metals and organic ligands together to enhance electrochemical performance, and opens up fresh avenues for the molecular design of CP-based electrode materials for advanced LIBs.

## Data Availability

Data are contained within the article and Supplementary Materials.

## Supplementary Materials

The following supporting information can be downloaded at: https://www.mdpi.com/article/10.3390/molecules28247993/s1, Figure S1. The 3D supramolecular structure of Co-PDA. Figure S2. The detailed FTIR spectra of Co-PDA and Ni-PDA before and after soaked in electrolyte. Figure S3. TGA curves of Co-DPA and Ni-PDA. Figure S4. SEM images of Co-PDA (a) and Ni-PDA (b) crystals and the corresponding EDS mapping images. Figure S5. Cycling stability at 1000 mA g^−1^ of Co-PDA and Ni-PDA. Figure S6. The electrochemical performance of the organic ligand H_2_PDA. Figure S7. (a) Cyclic voltammetry curves of Ni-PDA at 0.1 mV s^−1^. Figure S8. The corresponding XPS spectra of (a) Ni 2p, (b) O 1s, (c) C 1s, (d) N 1s and (e) Li 1s of Ni-PDA. Figure S9. Ex-situ FTIR spectra at different discharging and charging states of Ni-PDA. Figure S10. FTIR spectra of Co-PDA (a) and Ni-PDA (b) at selected states. Figure S11. Selected ex-situ PXRD patterns at the different states of Co-PDA (a) and Ni-PDA (b) electrodes during the first two cycles. Figure S12. GITT curves of Co-PDA (a) and Ni-PDA (b); (c) the calculated Li+ chemical diffusion coefficients based on GITT. Figure S13. (a) CV curves (A 3.0 V, B 1.25 V, C 0.82 V, D 0.01 V, E 1.46 V, F 1.88 V, and G 3.0 V) and the corresponding (b) impedance spectra of Ni-PDA at selected charge/discharge states; the relationship between *Z*′ and *ω*^−1/2^ of Co-PDA (c) and Ni-PDA; the calculated Li^+^ chemical diffusion coefficients based on EIS (e) and the calculated conductivity (f). Table S1. Data collection and processing parameters for Co-PDA. Table S2. Selected bond lengths (Å) and angles (°) for Co-PDA. Table S3. Selected bond lengths (Å) and angles (°) for Co-PDA. Table S4. The fitting result of equivalent circuit diagram of Co-PDA and Ni-PDA.
