# Supplementary material for: Facile Construction of Advanced 1D Metal-Organic Coordination Polymer for Efficient Lithium Storage
Source: Molecules. 2023 Dec 7;28(24):7993. doi: 10.3390/molecules28247993 (PMC10745800; doi:10.3390/molecules28247993)
Supplement: Supplementary file 1 [file molecules-28-07993-s001.zip › molecules-2757453-supplementary.docx]

**Supplementary Information**

**Facile Construction of Advanced 1D Metal-Organic Coordination Polymer for Efficient Lithium Storage**

**Jia Du ^1,2,^*, Xueguo Liu ^1^ and Bingke Li ^1^**

1. School of Biology and Chemical Engineering, Nanyang Institute of Technology, No.80, Changjiang Road, Nanyang 473004, China

2. Key Laboratory of Advanced Energy Materials Chemistry (Ministry of Education), College of Chemistry, Nankai University, Tianjin 300071, China

* Correspondence: 3132085@nyist.edu.cn


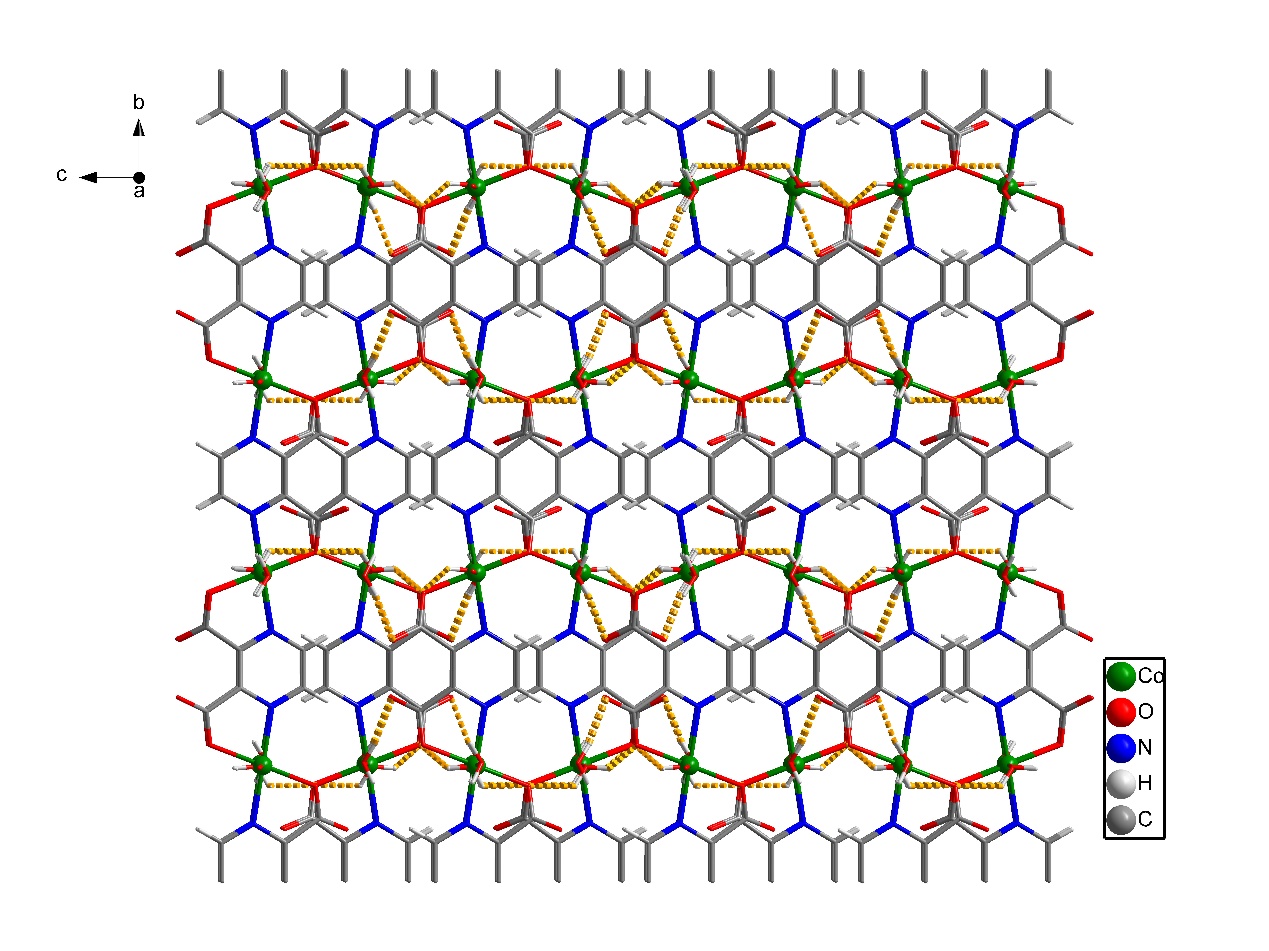


**Figure S1.** The 3D supramolecular structure of Co-PDA.


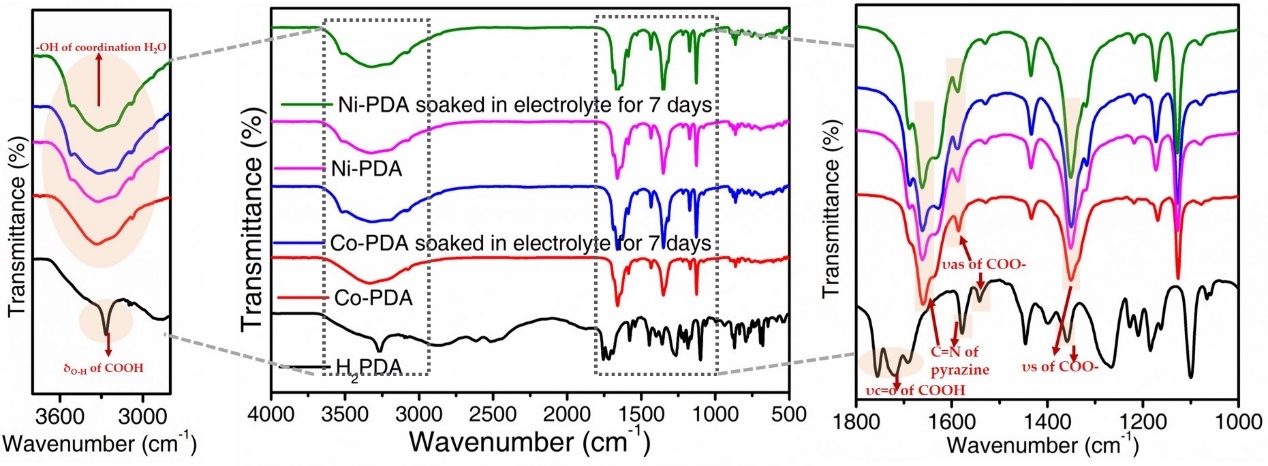


**Figure S2.** The detailed FTIR spectra of Co-PDA and Ni-PDA before and after soaked in electrolyte.


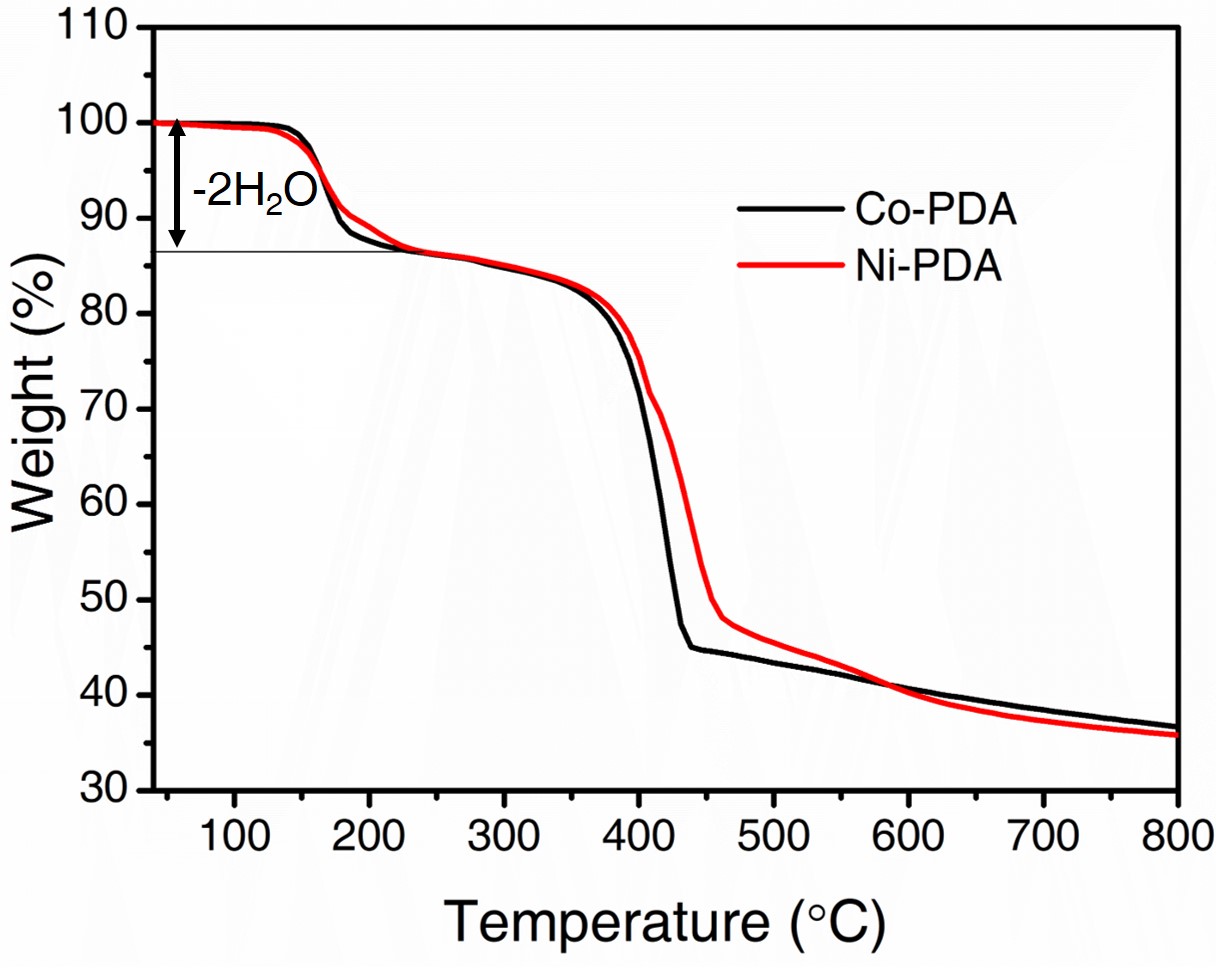


**Figure S3.** TGA curves of Co-DPA and Ni-PDA.


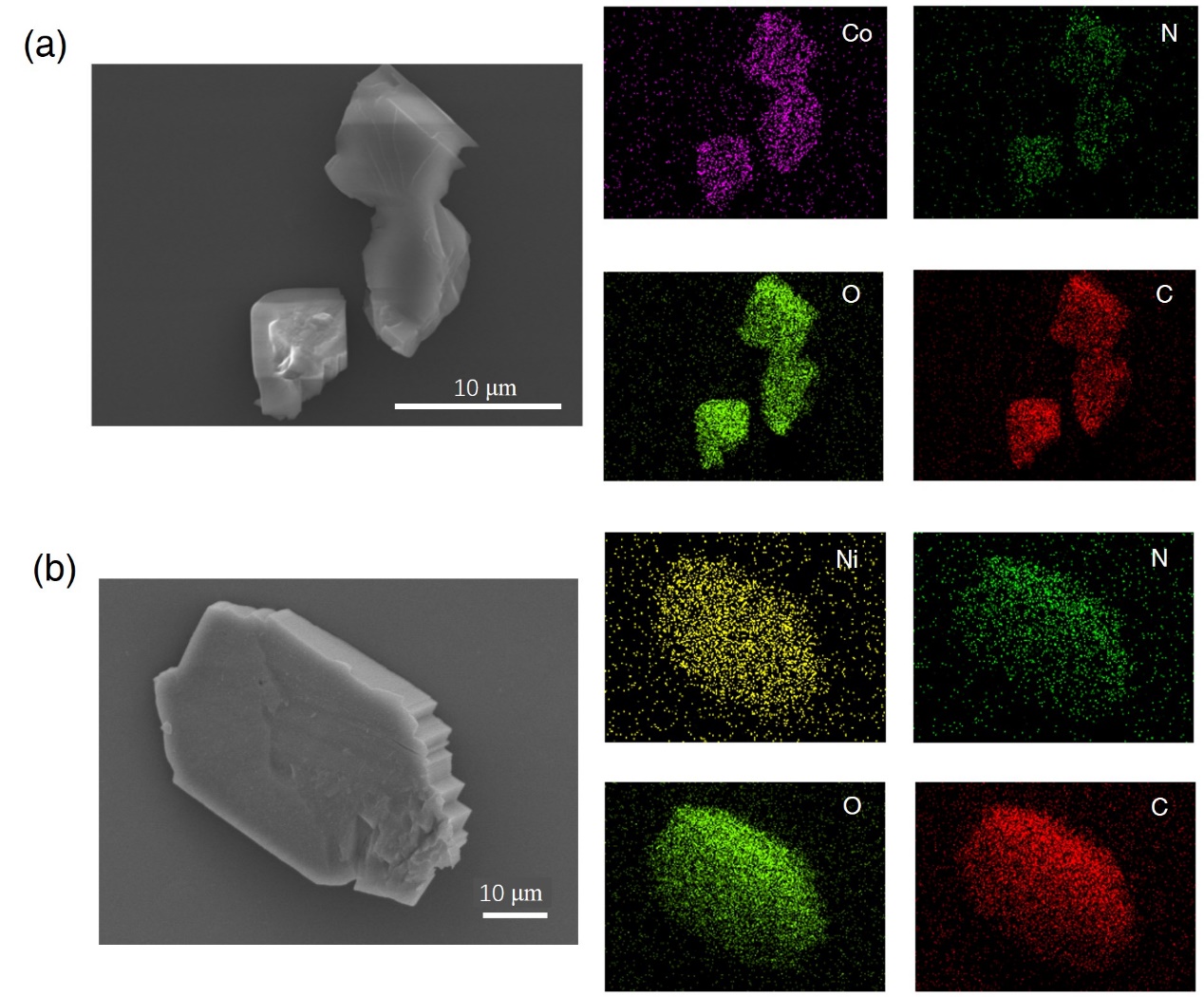


**Figure S4.** SEM images of Co-PDA (a) and Ni-PDA (b) crystals and the corresponding EDS mapping images.


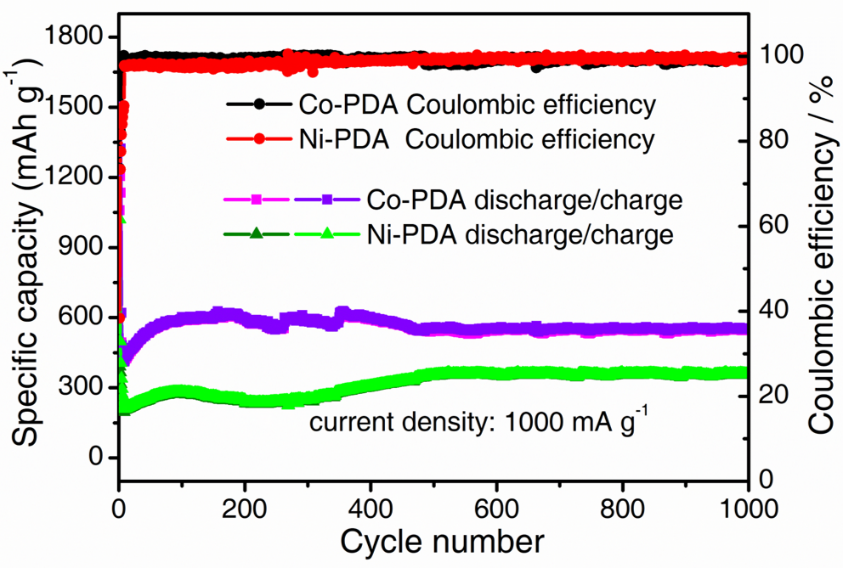


**Figure S5.** Cycling stability at 1000 mA g^-1^ of Co-PDA and Ni-PDA.


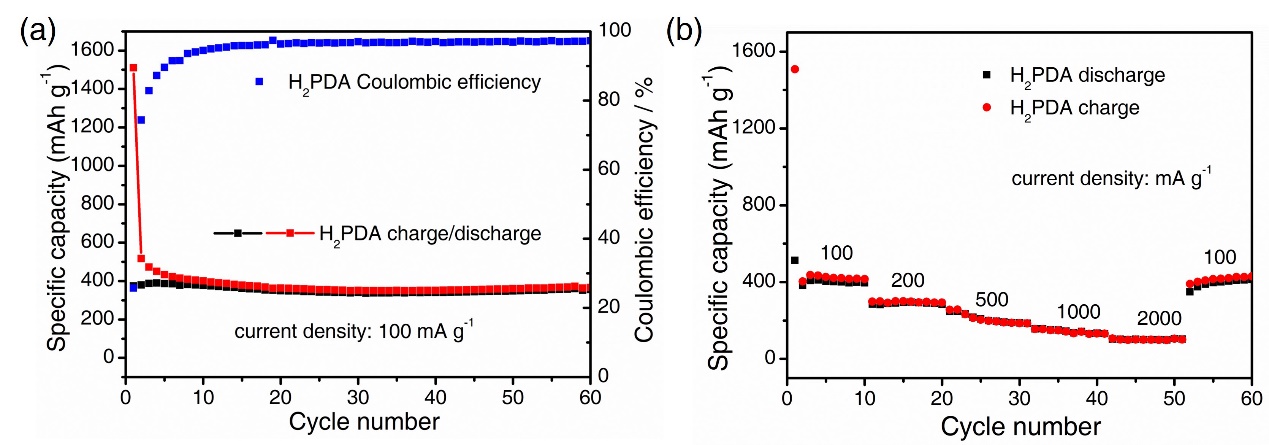


**Figure S6.** The electrochemical performance of the organic ligand H_2_PDA.


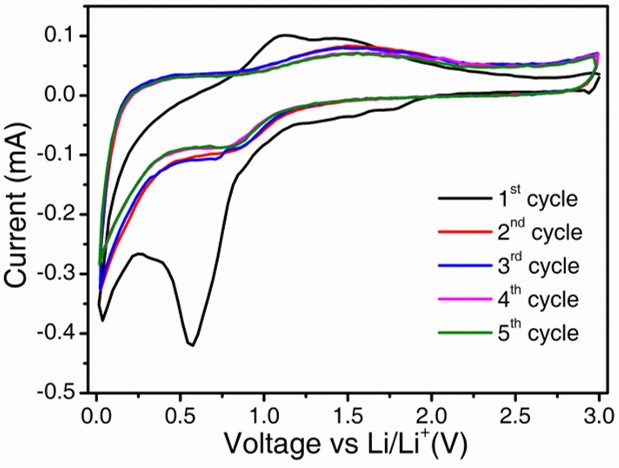


**Figure S7.** (a) Cyclic voltammetry curves of Ni-PDA at 0.1 mV s^−1^.


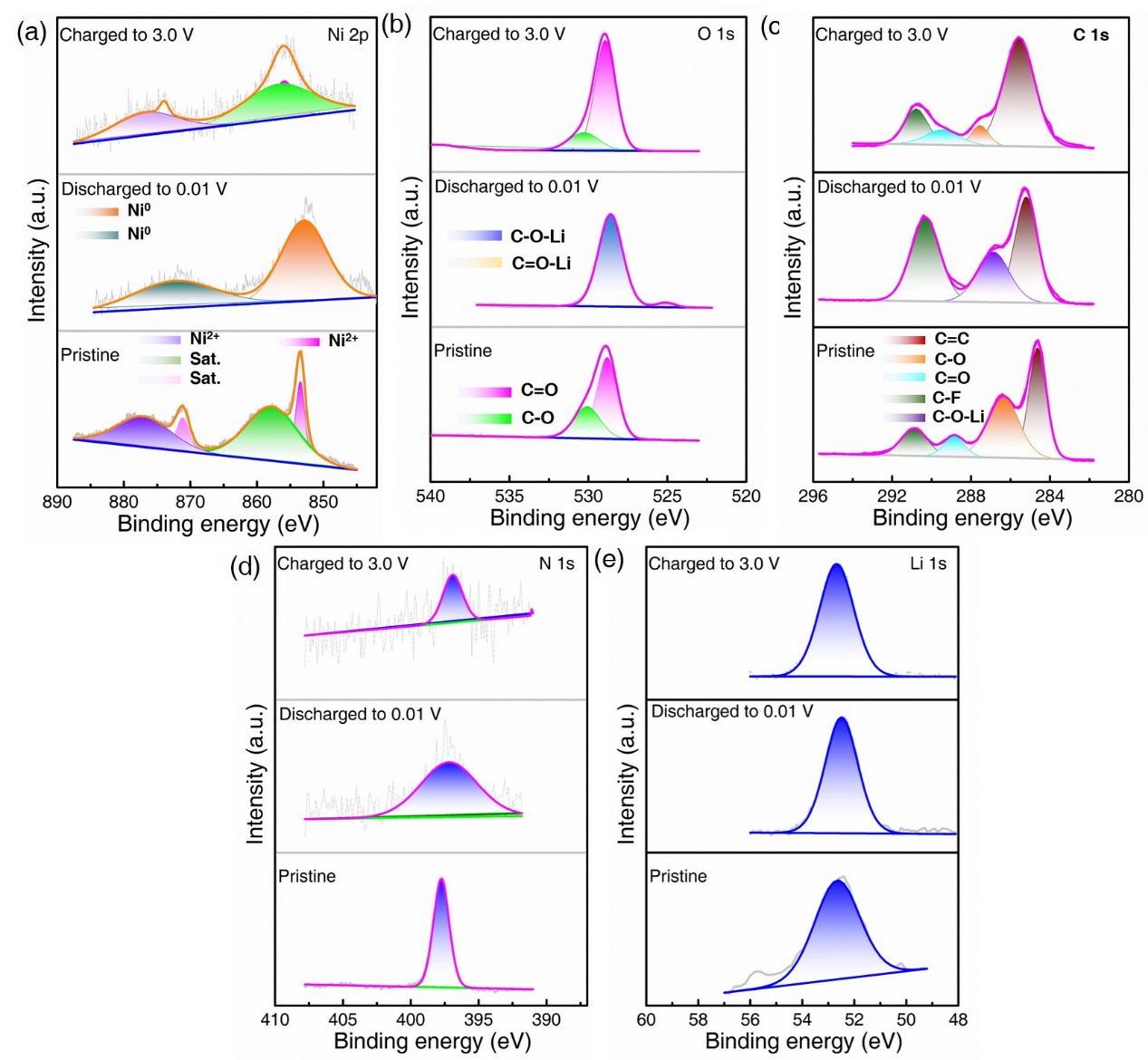


**Figure S8.** The corresponding XPS spectra of (a) Ni 2p, (b) O 1s, (c) C 1s, (d) N 1s and (e) Li 1s of Ni-PDA.


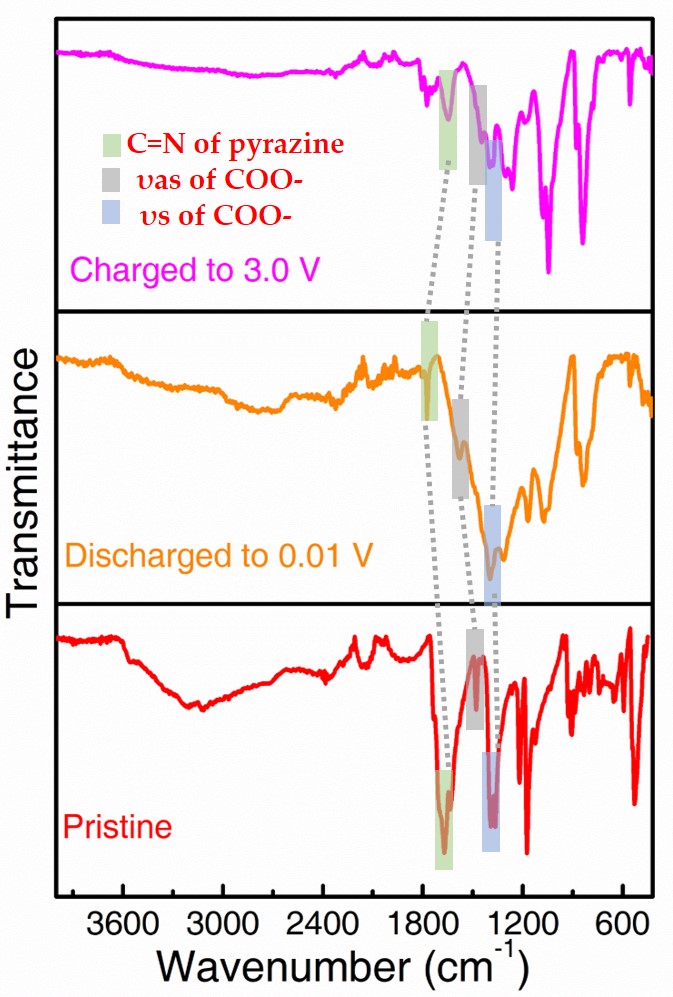


**Figure S9.** Ex-situ FTIR spectra at different discharging and charging states of Ni-PDA.


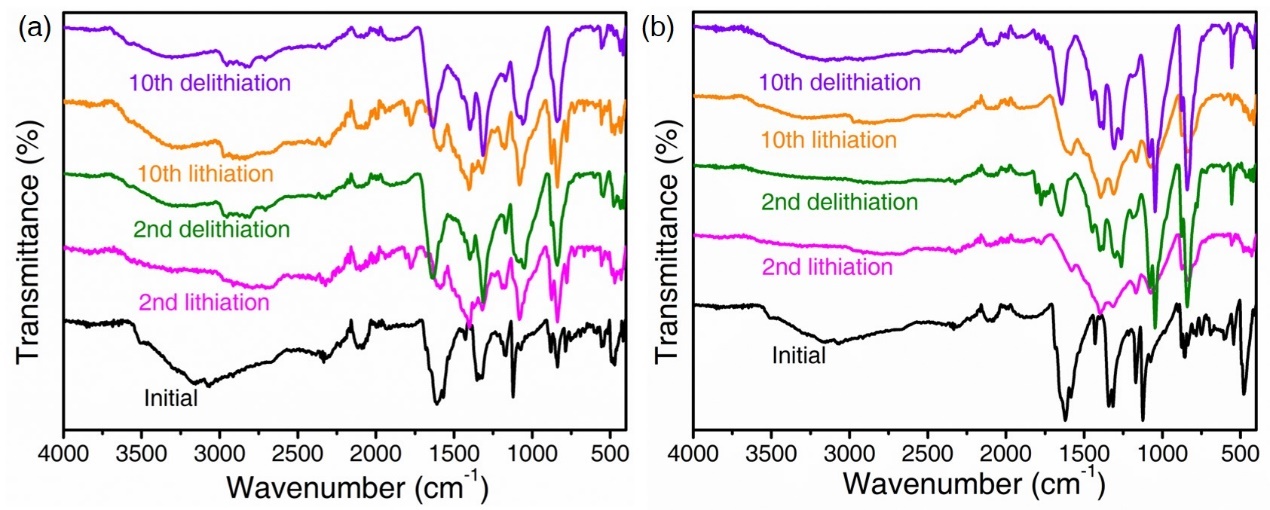


**Figure S10.** FTIR spectra of Co-PDA (a) and Ni-PDA (b) at selected states.


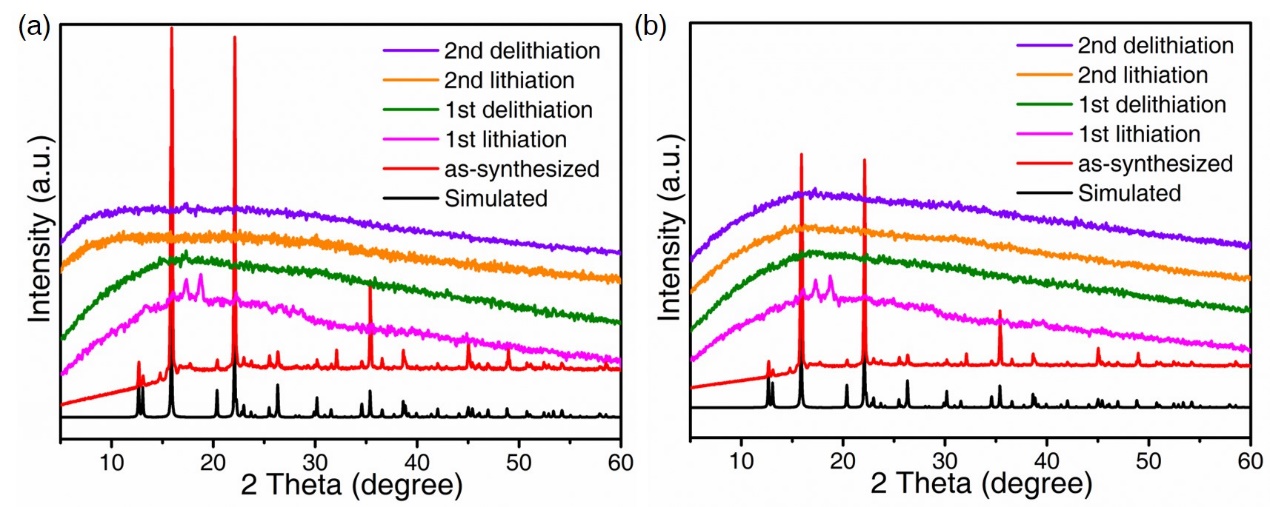


**Figure S11.** Selected ex-situ PXRD patterns at the different states of Co-PDA (a) and Ni-PDA (b) electrodes during the first two cycles.


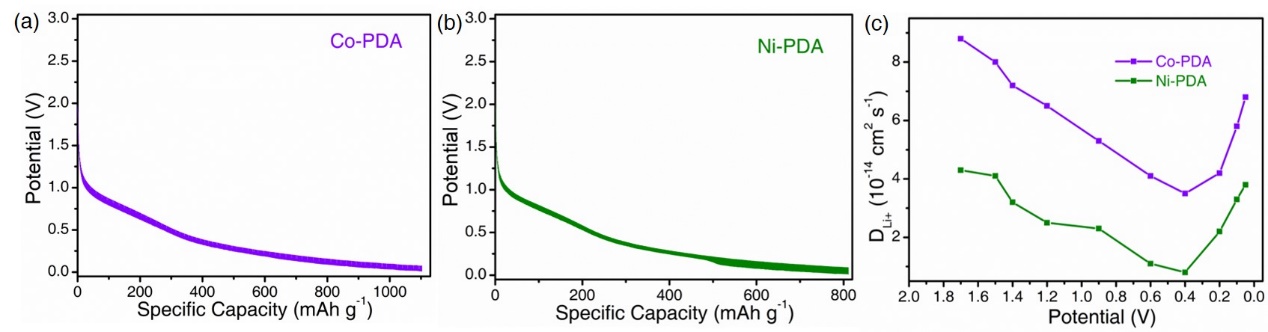


**Figure S12.** GITT curves of Co-PDA (a) and Ni-PDA (b); (c) the calculated Li^+^ chemical diffusion coefficients based on GITT.


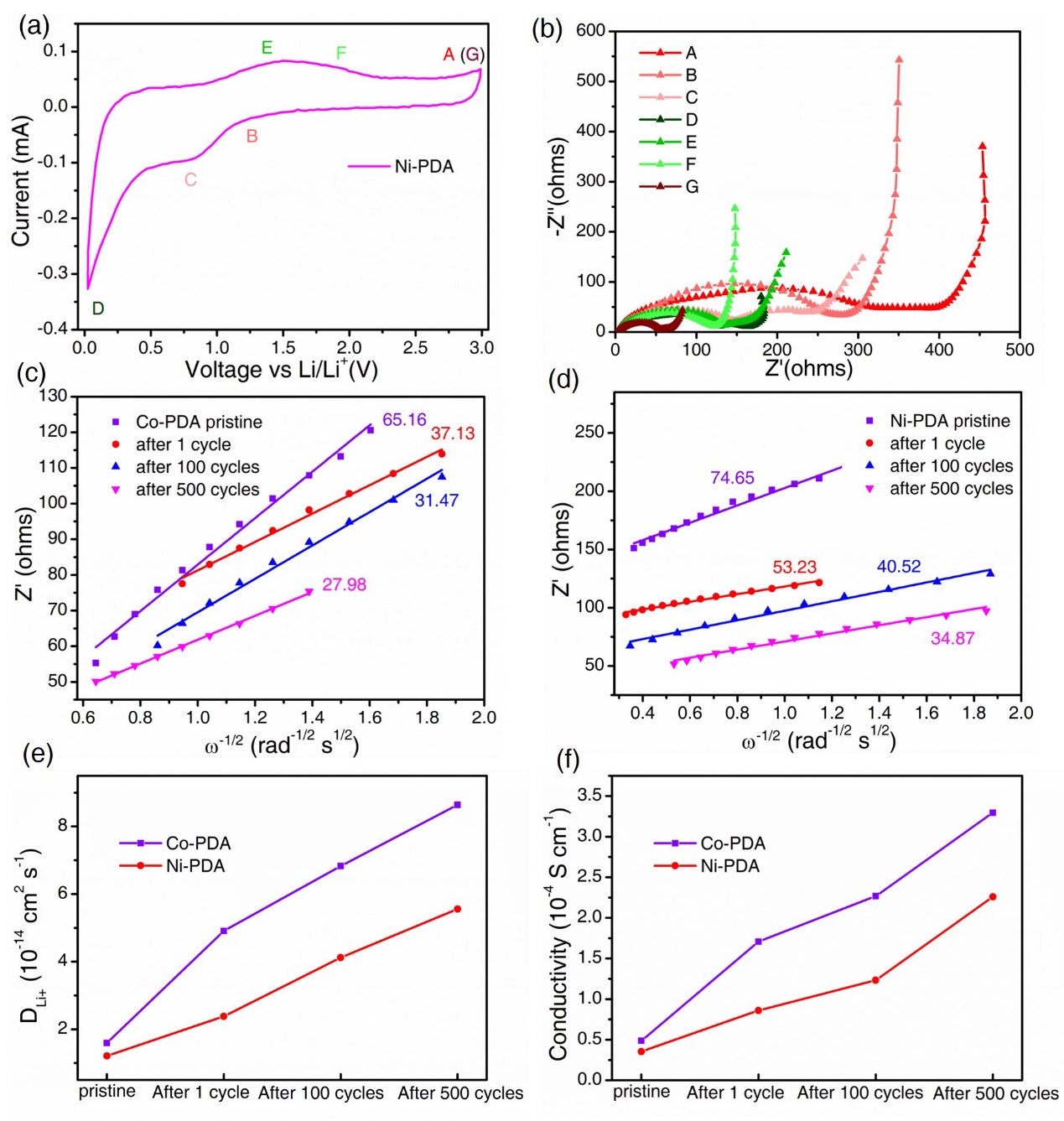


**Figure S13.** (a) CV curves (A 3.0 V, B 1.25 V, C 0.82 V, D 0.01 V, E 1.46 V, F 1.88 V, and G 3.0 V) and the corresponding (b) impedance spectra of Ni-PDA at selected charge/discharge states; the relationship between Z′ and ω^−1/2^ of Co-PDA (c) and Ni-PDA; the calculated Li^+^ chemical diffusion coefficients based on EIS (e) and the calculated conductivity (f).

**Table S1.** Data collection and processing parameters for Co-PDA.

|  | Co-PDA |
| --- | --- |
| formula | C_6_H_6_CoN_2_O_6_ |
| Formula weight | 261.06 |
| Temp, K | 298 |
| Crystal syst. | orthorhombic |
| Space group | Pcca |
| a/Å | 8.0591(3) |
| b/Å | 13.9861(5) |
| c/Å | 15.4992(7) |
| *α*, deg | 90 |
| *β*, deg | 90 |
| *γ*, deg | 90 |
| V, Å^3^ | 1747.00(12) |
| Z | 8 |
| Dc, g/cm^3^ | 1.985 |
| μ/mm^-1^ | 1.977 |
| Data/parameters | 2326/138 |
| F(000) | 1048.0 |
| Crystal size/mm^3^ | 0.05 × 0.04 × 0.02 |
| 2*θ* range (°) | 5.256 to 58.256 |
| Index ranges | -10 ≤ h ≤ 11, -18 ≤ k ≤ 17, -21 ≤ l ≤ 10 |
| Obs refins | 8273 |
| R_int_/GooF on F^2^ | 0.0308/1.014 |
| R_1_, *w*R_2_ [I>=2σ (I)] | 0.0394, 0.1180 |
| R_1_, *w*R_2_ (all data) | 0.0545, 0.1286 |
| max/min, e Å^-3^ | 0.61/-0.63 |

**Table S2.** Selected bond lengths (Å) and angles (º) for Co-PDA.

| Atom-atom | Length/Å | Atom-atom-atom | Angle/˚ | Atom-atom-atom | Angle/˚ |
| --- | --- | --- | --- | --- | --- |
| Co(1)−O(1) | 2.0600(15) | O(2)−Co(1)−N(6) | 101.70(7) | N(8)−Co(1)−N(6) | 177.50(7) |
| Co(1)−O(2) | 2.0539(16) | O(2)−Co(1)−O(7) | 89.03(6) | O(9)−Co(1)−O(2) | 90.37(7) |
| Co(1)−O(7) | 2.077(2) | O(2)−Co(1)−N(8) | 77.98(7) | O(9)−Co(1)−O(4) | 91.65(7) |
| Co(1)−O(9) | 2.049(2) | O(4)−Co(1)−O(2) | 177.98(6) | O(9)−Co(1)−N(6) | 87.36(7) |
| Co(1)−N(6) | 2.1577(19) | O(4)−Co(1)−N(6) | 78.41(7) | O(9)−Co(1)−O(7) | 179.31(7) |
| Co(1)−N(8) | 2.151(2) | O(4)−Co(1)−O(7) | 88.95(6) | O(9)−Co(1)−N(8) | 90.16(7) |
|  |  | O(7)−Co(1)−N(6) | 92.44(7) | O(7)−Co(1)−N(8) | 90.03(7) |
|  |  | O(4)−Co(1)−N(8) | 102.00(7) |  |  |

**Table S3.** Selected bond lengths (Å) and angles (º) for Co-PDA.

| Donor−H…Acceptor | D−H (Å) | H…A (Å) | D…A (Å) | D−H…A(°) |
| --- | --- | --- | --- | --- |
| O(7)−H(A)…O(4) | 0.86 | 2.19 | 2.725(3) | 120 |
| O(7)−H(B)…O(2) | 0.86 | 1.88 | 2.706(2) | 161 |
| O(9)−H(C)…O(3) | 0.86 | 2.21 | 2.815(2) | 127 |
| O(9)−H(C)…O(4) | 0.86 | 2.27 | 3.060(3) | 153 |
| O(9)−H(D)…O(5) | 0.86 | 1.99 | 2.829(2) | 166 |

**Table S4.** The fitting result of equivalent circuit diagram of Co-PDA and Ni-PDA.

| State | R_sol_ | F | R_ct_ | W |
| --- | --- | --- | --- | --- |
| Co-PDA pristine | 3 | 3.046×10^-6^ | 272 | 0.0024 |
| Co-PDA after 1 cycle | 5 | 1.247×10^-7^ | 77 | 0.002799 |
| Co-PDA after 100 cycles | 5 | 5.167×10^-6^ | 58 | 0.004089 |
| Co-PDA after 200 cycles | 4 | 2.33×10^-6^ | 40 | 0.007057 |
| Ni-PDA pristine | 20 | 3.93×10^-6^ | 374 | 0.006999 |
| Ni-PDA after 1 cycle | 9 | 5.477×10^-6^ | 154 | 0.009167 |
| Ni-PDA after 100 cycles | 6 | 5.414×10^-6^ | 107 | 0.01682 |
| Ni-PDA after 200 cycles | 4 | 4.062×10^-6^ | 58 | 0.01176 |
